# First and Second Dissociation Enthalpies in Bi-Component Crystals Consisting of Maleic Acid and L-Phenylalanine

**DOI:** 10.3390/molecules26185714

**Published:** 2021-09-21

**Authors:** Inês O. Feliciano, Daniela P. Silva, M. Fátima M. Piedade, Carlos E. S. Bernardes, Manuel E. Minas da Piedade

**Affiliations:** 1 Centro de Química Estrutural, Faculdade de Ciências, Universidade de Lisboa, 1749-016 Lisboa, Portugal; Idfeliciano@fc.ul.pt (I.O.F.); cebernardes@fc.ul.pt (C.E.S.B.); 2 Departamento de Química e Bioquímica, Faculdade de Ciências, Universidade de Lisboa, 1749-016 Lisboa, Portugal; mdpiedade@fc.ul.pt; 3 Centro de Química Estrutural, Instituto Superior Técnico, Universidade de Lisboa, 1049-001 Lisboa, Portugal; Daniela.filipa.2710@hotmail.com

**Keywords:** multicomponent crystal, energetics, solution calorimetry, differential scanning calorimetry, thermogravimetry, X-ray diffraction

## Abstract

The energetics of the stepwise dissociation of a A:B_2_ bi-component crystal, according to A:B_2_(cr) → A:B(cr) + B(cr) and A:B(cr) → A(cr) + B(cr), was investigated using MA:Phe_2_ and MA:Phe (MA = maleic acid; Phe = L-phenylalanine) as model systems. The enthalpy changes associated with these sequential processes and with the overall dissociation reaction A:B_2_(cr) → A(cr) + 2B(cr) were determined by solution calorimetry. It was found that they are all positive, indicating that there is a lattice enthalpy gain when MA:Phe_2_ is formed, either from the individual precursors or by adding Phe to MA:Phe. Single-crystal X-ray diffraction (SCXRD) analysis showed that MA:Phe_2_ is best described as a protic salt containing a maleate anion (MA^−^) and two non-equivalent L-phenylalanine units, both linked to MA^−^ by NH···O hydrogen bonds (H-bond): one of these units is protonated (HPhe^+^) and the other zwitterionic (Phe^±^). Only MA^−^ and HPhe^+^ molecules are present in the MA:Phe lattice. In this case, however, NH···O and OH···O H-bonds are formed between each MA^−^ unit and two HPhe^+^ molecules. Despite these structural differences, the enthalpy cost for the removal of the zwitterionic Phe^±^ unit from the MA:Phe_2_ lattice to yield MA:Phe is only 0.9 ± 0.4 kJ mol^−1^ higher than that for the dissociation of MA:Phe, which requires a proton transfer from HPhe^+^ to MA^−^ and the rearrangement of L-phenylalanine to the zwitterionic, Phe^±^, form. Finally, a comparison of the dissociation energetics and structures of MA:Phe and of the previously reported glycine maleate (MA:Gly) analogue indicated that parameters, such as the packing coefficient, density, hydrogen bonds formed, or fusion temperature, are not necessarily good descriptors of dissociation enthalpy or lattice enthalpy trends when bi-component crystals with different molecular composition are being compared, even if the stoichiometry is the same.

## 1. Introduction

Multi-component organic crystals have been known of since at least 1844, when Wöhler synthesized quinhydrone from *p*-quinone and hydroquinone [1]. Interest in these materials was renewed in recent years, after it was widely recognized that the incorporation of two or more different molecules in the same crystal could open a variety of opportunities for product innovation [2] and patenting [3] in industrial sectors such as dyes [4], agrochemicals [5], optics [6,7], energetic materials [8,9,10], and pharmaceuticals [11,12,13,14,15,16,17,18]. Thus, for example, energetic materials made of bi-component crystals with better stability than their individual precursors have been reported [8,9,10], and medicines based on bi-component crystals (API-CF) consisting of an active pharmaceutical ingredient (API) and a pharmaceutically acceptable co-former (CF) or two APIs (API–API) have been marketed or are in clinical development [19,20]. In the pharmaceutical area, where the main driving force for research on multi-component crystals currently resides, the goal is typically the improvement and modulation of properties that need to be strictly controlled to warrant the optimal and reproducible performance of a drug (e.g., chemical and physical stability, tabletability, hygroscopicity, solubility, dissolution rate, bioavailability) [11,12,13,14,15,16,17,18], or the achievement of multimodal therapy [21,22]. But it may also be the inhibition of co-crystallization to avoid loss of efficacy of a fixed dose combination of two APIs, as in the case of the olmesartan medoxomil and hydrochlorothiazide formulation used to treat hypertension [23].

A central issue for *a priori* guiding the synthesis of multi-component crystals, or the inhibition of their formation, is a reasonable understanding of the structure–energetics relationships that determine their thermodynamic stability towards dissociation into the precursors. These relationships are, however, still poorly understood at molecular level (e.g., the importance of hydrogen- or halogen-bond interactions and the packing efficiency for the stability of multi-component crystals is not clear) [24,25], and their illumination requires a sufficiently large body of structural and thermodynamic information.

Thermodynamic stability is most appropriately assessed in terms of the Gibbs energy change, $\Delta_{r}G_{m}$, of plausible dissociation reactions [26], stability implying $\Delta_{r}G_{m}$ > 0 for the decomposition process considered. Nevertheless, it seems that the stability of multi-component crystals is most often controlled by enthalpic rather than entropic contributions to $\Delta_{r}G_{m}$, meaning that the dominant factor is the lattice energy loss associated with their dissociation into the individual precursors. This is supported by two wide-scope studies on the energetics of co-crystals, one based on periodic density functional theory (DFT) calculations [25] and the other on an empirical estimation scheme [24], suggesting a dominant enthalpy term in 70–95% of cases. This tendency is expected to be even more valid in the case of protic organic salts, where partial or complete proton transfer between the co-formers leads to the development of an enhanced coulombic contribution that is likely to reinforce the multi-component crystal lattice energy [27]. The generality of this hypothesis still needs, however, confirmation, given that the energetics of a dissociation process is not exclusively determined by the reactant considered but also depends on the products formed.

Experimental data are, of course, essential for the understanding of structure–energetics relationships in multi-component crystals, and for the development and validation of theoretical approaches such as those mentioned above. However, in contrast with the large body of available structural information [28], studies where the thermodynamic stability of multi-component crystals has been quantitatively assessed in terms of enthalpy or Gibbs energy changes are scarce. Representative examples include systems such as the already mentioned hydroquinone:quinhydrone [29] and, more recently, celecoxib:nicotinamide [30], flubendazole:maleic acid [31], fumaric acid:glycine [27], maleic acid:glycine [27], sulfamethazine:salicylic acid [32,33], itraconazole:4-aminobenzoic acid [34], and itraconazole:4-hydroxybenzamide [34].

One aspect that has been little explored from an experimental perspective is the influence of stoichiometry on the energetics of multi-component crystals. There have been reports showing that in the case of the carbamazepine:*p*-aminobenzoic acid system, there is no correlation between the dissolution rate and the stoichiometry (1:1, 2:1, or 4:1) of multicomponent crystals [35], demonstrating that in the same case, the preference for a 2:1 or 1:1 stoichiometry can be tuned by changing the composition of the solution in equilibrium with the solid phase [36], or suggesting (based on an empirical estimation scheme) that, in general, the stoichiometry with a higher fusion temperature corresponds to a higher stability [24]. We are not aware, however, of experimental studies where the influence of stoichiometry on the thermodynamic stability of multicomponent crystals relative to their precursors in the absence of solvent has been investigated. Here, we address this problem using a model system consisting of two-component crystals composed of maleic acid (MA) and L-phenylalanine (Phe) with 2:1 and 1:1 stoichiometries. We also introduce the concepts of multi-component crystal mean and stepwise dissociation enthalpy, as measures of the overall interaction strength determining stability.

If bi-component crystals are regarded as a supermolecules composed of A and B species, then the standard molar enthalpies of the reactions:

A:B_2_(cr) → A:B(cr) + B(cr) (1)

A:B(cr) → A(cr) + B(cr)(2)

$\Delta_{r}H_{m}^{o}\left(1\right)$ and $\Delta_{r}H_{m}^{o}\left(2\right)$, respectively, can be viewed as the analogous of stepwise bond dissociation enthalpies in molecules [26] and denoted by $DH_{1}^{o}{\left(A:B\right)}_{\mathrm{cr}}$ and $DH_{2}^{o}{\left(A:B\right)}_{\mathrm{cr}}$, where the subscript “_cr_” indicates that the dissociation refers to the crystalline state. By the same token, if reaction:

A:B2(cr) → A(cr) + 2B(cr)(3)

is considered, then $0.5\Delta_{r}H_{m}^{o}\left(3\right)$ is similar to a mean bond dissociation enthalpy and can be represented by $\bar{DH^{o}}{\left(A:B\right)}_{\mathrm{cr}}$. Needless to say, that, in contrast with bond dissociation enthalpies, the $DH^{o}{\left(A:B\right)}_{\mathrm{cr}}$ values cannot be simply associated with the breaking of specific covalent bonds, but reflect the overall change in intermolecular interactions that occur when a multi-component crystal dissociates into its precursors in one or more steps. Nevertheless, positive $DH^{o}{\left(A:B\right)}_{\mathrm{cr}}$ values indicate that there is a loss in lattice enthalpy when the multi-component crystal is dissociated or, in other words, on enthalpic grounds the multi-component crystal is more stable than its dissociation products. The present study explored the possible relationships between dissociation enthalpies, lattice enthalpies, and structural descriptors such as packing coefficient and H-bond patterns.

## 2. Results and Discussion

### 2.1. Synthesis

Two bi-component crystals composed by maleic acid and L-phenylalanine with 1:1 (MA:Phe) and 1:2 (MA:Phe_2_) stoichiometries were synthesized using mechanochemistry. Solvent free grinding was used and the precursor samples corresponded to the form I polymorphs of MA (CSD Refcode: MALIAC11) [28,37] and Phe (CSD Refcode: QQQAUJ06) [28,38]. Indexation of the X-ray powder diffraction pattern (see Supplementary Materials) indicated that the produced MA:Phe sample was compatible with the crystal structures of previously reported phenylalanine maleate forms obtained by crystallization from water, which differ only by the fact that the configuration of phenylalanine is inverted. One of these structures refers to 25 K (EDAXIQ03 [28,39]) and the other to 293 K (CSD Refcode: EDAXIQ) [28,40]. MA:Phe_2_ was prepared for the first time. Analysis of the jar contents at different grinding times, by X-ray powder diffraction, suggested that MA:Phe is an intermediate of the MA:Phe_2_ formation (see Figure S1 in the Supplementary Materials). Crystals of MA:Phe_2_ suitable for a molecular and crystal structure determination by single-crystal X-ray diffraction were grown by solvent evaporation. The initial solution consisted of 25 mg of the synthesized material in 2 mL of water. After 2–3 weeks at ambient temperature (293 ± 2 K), transparent parallelepipedal crystals were obtained.

### 2.2. Structure

Single-crystal X-ray diffraction analysis of MA:Phe_2_ crystals carried out at 168 ± 2 K and 293 ± 2 K showed no significant changes in the supramolecular structure with temperature. Therefore, only the 293 ± 2 K results are discussed here, since they correspond to a temperature which is closer to the reference temperature of the thermodynamic data (298 K).

**Figure 1:**
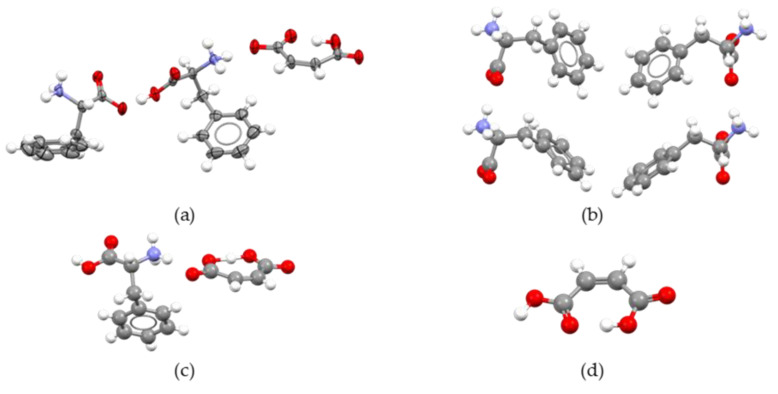
Mercury 3.10.1 (Build 290188) [41] diagram of the asymmetric units of: (**a**) MA:Phe_2_ (this work, the thermal ellipsoids of C, N, and O atoms were drawn at 50% probability); (**b**) L-phenylalanine (polymorph I, CSD Refcode: QQQAUJ06 [28,38]); (**c**) MA:Phe (CSD Refcode: EDAXIQ) [28,40]); (**d**) maleic acid (polymorph I, CSD Refcode: MALIAC11, [28,37]).

The asymmetric unit of MA:Phe_2_ is compared in Figure 1 with those of MA:Phe [28,40] and of the precursors MA(form I) [28,37] and Phe (form I) [28,38] at 293 K. It should be noted that although the absolute configuration of phenylalanine in the MA:Phe structure reported at 293 K [28,40] is inverted relative to that at 25 K [28,39], no significant differences are noted in terms of the molecular packing between both structures (see Figure S2 in the Supplementary Materials). Figure 1a shows that the formation of MA:Phe_2_ involves a proton transfer from maleic acid to one of the L-phenylalanine molecules, with the second L-phenylalanine remaining zwitterionic as in the pure reactant form (Figure 1b). The crystal lattice, therefore, contains a combination of three different species: a maleate anion (MA^−^), a L-phenylalanine cation (HPhe^+^), and a zwitterionic L-phenylalanine (Phe^±^). The proton transfer from maleic acid to phenylalanine is also present in the case of MA:Phe (Figure 1c). Thus, from a molecular structure perspective, the two bi-component crystals investigated in this work essentially differ by the presence of a zwitterionic L-phenylalanine molecule in MA:Phe_2_, which is absent in MA:Phe. The asymmetric unit of pure maleic acid (Figure 1d) consists of a single neutral molecule that retains the same type of intramolecular O−H∙∙∙O hydrogen bond of the anionic forms present in MA:Phe and MA:Phe_2_.

**Figure 2:**
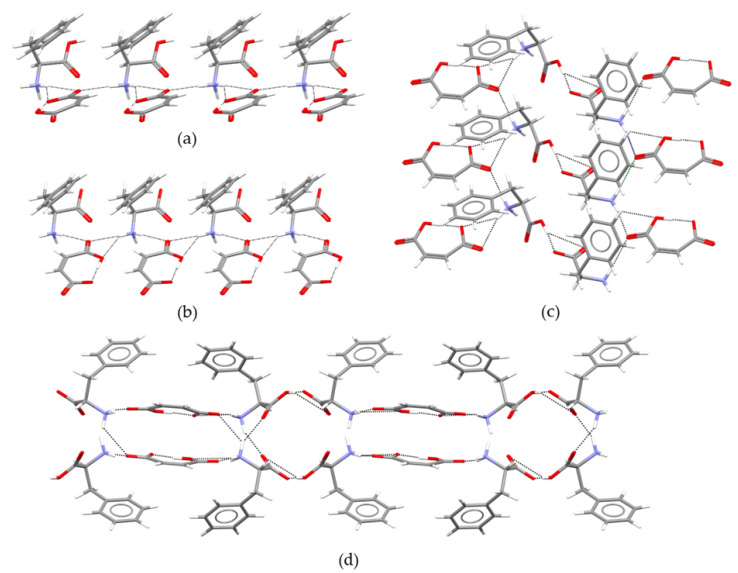
Packing motifs of MA:Phe_2_: (**a**) $C_{2}^{1}(4)$ chain consisting of cationic and anionic units, HPhe^+^ − MA^−^ − HPhe^+^; (**b**) $C_{2}^{1}(4)$ chain composed of zwitterionic and anionic units, Phe^±^ − MA^−^ − Phe^±^; (**c**) 2D packing motif in the *bc* plane; (**d**) 3D crystal packing projected in the *ac* plane.

From a packing point of view, two 1D chain motifs, both of the $C_{2}^{1}(4)$ type, can be recognized in MA:Phe_2_. The first one (Figure 2a) is composed of alternating cationic and anionic units and is sustained by bifurcated (d_NH∙∙∙O_ = 2.27(3) Å and 2.41(3) Å) and single (d_NH∙∙∙O_ = 2.02(4) Å) H-bonds between the ammonium group of HPhe^+^ and the carboxylate group of MA^−^. The second one (Figure 2b) is formed by maleate anions and zwitterionic L-phenylalanine molecules, −Phe^±^ − MA^−^ − Phe^±^ −, and also involves bifurcated (d_NH∙∙∙O_ = 2.11(4) Å and 2.55(3)) and single (d_NH∙∙∙O_ = 2.26(3) Å) H-bonds of N−H∙∙∙O type. Both chains propagate along the *b*-axis, and they link to each other through a bifurcated O−H∙∙∙O hydrogen bond (d_OH∙∙∙O_ = 1.28(3) Å and 2.54(4) Å) involving the carboxylic and carboxylate groups of adjacent HPhe^+^ and Phe^±^ molecules, establishing a 2D sheet (Figure 2c). The final 3D packing (Figure 2d) is completed by staking these 2D sheets along the *a*-axis. The stacking is ensured by two N−H∙∙∙O hydrogen bonds: one between the carboxylate group of a maleate molecule and the ammonium group of a zwitterionic L-phenylalanine (d_NH∙∙∙O_ = 2.39(3) Å) and the other between the carboxylic group of a cationic L-phenylalanine and the ammonium group of a zwitterionic L-phenylalanine (d_NH∙∙∙O_ = 2.08(4) Å).

**Figure 3:**
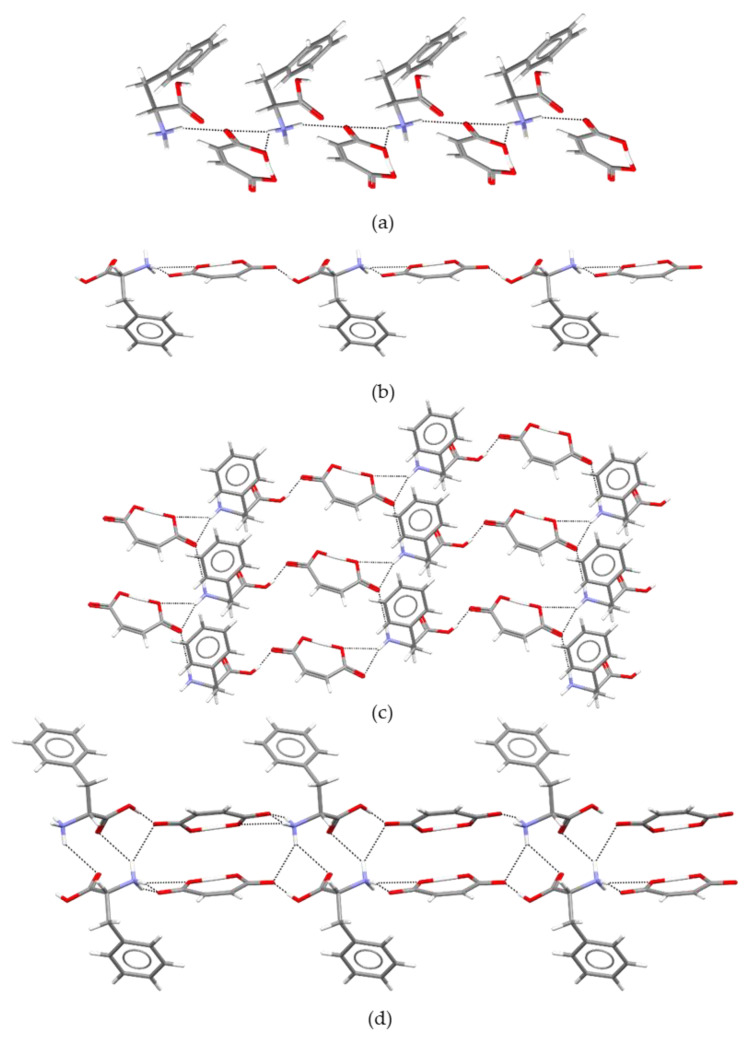
Packing motifs identified in the previously reported MA:Phe structure [40]: (**a**) $C_{2}^{1}(4)$ chain; (**b**) $C_{2}^{1}(11)$ chain; (**c**) 2D packing motif in the *bc* plane; (**d**) 3D packing projected in the *ac* plane.

The packing architecture of MA:Phe (Figure 3) is, in some respects, similar to that of MA:Phe_2_. The molecules are also organized in $C_{2}^{1}(4)$ chains defining −HPhe^+^−MA^−^−HPhe^+^− one-dimensional patterns that propagate along the *b*-axis (Figure 3a). These chains are likewise sustained by bifurcated (d_NH∙∙∙O_ = 2.20 Å and 2.29 Å) and single (d_NH∙∙∙O_ = 2.10 Å) N−H∙∙∙O hydrogen bonds between the ammonium group of HPhe^+^ and the COO···OOC framework of MA^−^ (the exact position of the H atom sustaining the intramolecular OH···OC bond in maleic acid is not known in the reported MA:Phe structure [40]). In this case, however, a second 1D motif of −HPhe^+^ − MA^−^ − HPhe^+^− type can be found (Figure 3b). This corresponds to a $C_{2}^{2}(11)$ chain, that propagates along the *c*-axis, sustained by the same N−H∙∙∙O (d_NH∙∙∙O_ = 2.20 Å and 2.29 Å) bifurcated hydrogen bonds belonging to the $C_{2}^{1}(4)$ chain and by O−H∙∙∙O hydrogen bonds (d_OH∙∙∙O_ = 1.74 Å) involving the carboxylic group of HPhe^+^ and an carboxylate oxygen of MA^−^. The two chains with a common hydrogen bond define the 2D pattern as shown in Figure 3c. The 3D framework is built by stacking the 2D sheets along the *a*-axis by means of an extra N−H∙∙∙O bifurcated hydrogen bond (d_NH∙∙∙O_ = 2.25 Å and 2.45 Å) between MA^−^ and two HPhe^+^ units (Figure 3d).

**Figure 4:**
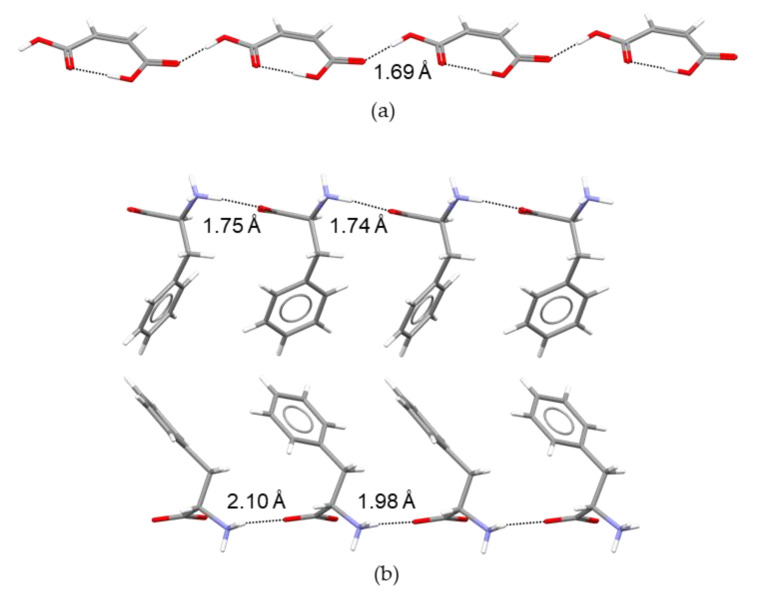
(**a**) The $C_{1}^{1}(7)$ chain 1D motif present in maleic acid packing and (**b**) the two $C_{1}^{1}(10)$ chains typical of L-phenylalanine packing.

The molecular structure differences observed in the unit cells of pure maleic acid and L-phenylalanine compared to those of MA:Phe and MA:Phe_2_ (Figure 1) are also reflected by the packing arrangements. This can be illustrated by the 1D motifs in Figure 4. As shown in Figure 4a, the neutral MA molecules are linked to each other by O−H∙∙∙O hydrogen bonds (*d*_OH∙∙∙O_ = 1.69 Å) defining a $C_{1}^{1}(7)$ linear chain. In the case of L^−^.

Phenylalanine, two $C_{2}^{2}(10)$ chains sustained by N−H∙∙∙O hydrogen bonds are present (Figure 4b). Each one of them involves a pair of non-equivalent zwitterionic molecules separated by different distances (top: *d*_NH∙∙∙O_ = 1.74 Å and 1.75 Å; bottom: *d*_NH∙∙∙O_ = 1.98 Å and 2.10 Å).

The volumetric features of MA:Ph, MA:Phe_2_, and their precursors are summarized in Table 1. The data evidence two clear trends that reflect the relationship between molecular size/shape and close packing: (i) all four packing coefficients (*k*) are in the 0.65–0.77 range, which is typical of the close packing range found for spherical or ellipsoid objects [42]; (ii) the densities of MA:Phe and MaPhe_2_ are within those of MA (high limit) and Phe (low limit) and become closer to the low limit as the number of Phe units in the crystal lattice increases.

### 2.3. Thermal Analysis

**Figure 5:**
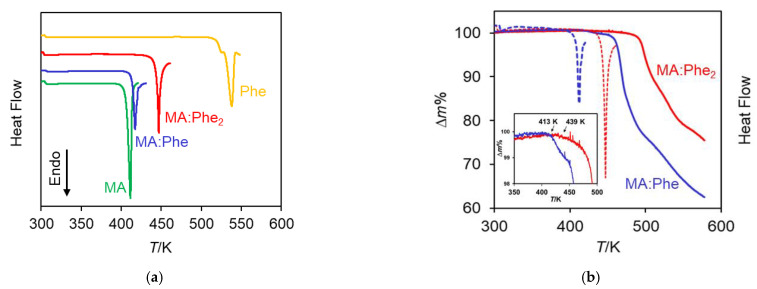
(**a**) Typical DSC results for MA:Phe, MA:Phe_2_, MA(cr I), and Phe(cr I): (**b**) overlay of DSC and TG curves for MA:Phe and MA:Phe_2_ (the inset shows the beginning of the mass loss events). All experiments were carried out at a heating rate of 5 K∙min^−1^.

The thermal behaviors of MA:Phe and MA:Phe_2_ were investigated by differential scanning calorimetry (DSC) and thermogravimetry (TG) at a heating rate of 5 K⋅min^−1^. For comparison purposes, DSC experiments were also carried out on the MA(cr I) and Phe(cr I) precursors. As shown in Figure 5a, no phase transitions other than fusion were noted in the DSC curves obtained for all species, between 298 K and the fusion temperature. The values of the onset (*T*_on_) and maximum (*T*_max_) temperatures of the fusion peaks are summarized in Table 2, where the assigned uncertainties correspond to twice the standard error of the mean of the number of replicates given in parenthesis. Only a single run was made for Phe since sublimation of the compound followed by melting and formation of gaseous decomposition products led to crucible leakage. Moreover, the enthalpy of fusion, $\Delta_{\mathrm{fus}}H_{m}^{o}$, could only be determined for MA(cr I), because in all other cases fusion was accompanied by thermal decomposition. This was evidenced by the presence of brownish residues inside the crucibles at the end of the experiments with MA:Phe, MA:Phe_2_, and Phe(cr I).

Two major conclusions can be drawn from Table 2: (i) the fusion temperatures of MA:Phe (*T*_on_ = 415.8 ± 0.6 K) and MA:Phe_2_ (*T*_on_ = 444.6 ± 0.6 K) fall within those of pure MA (*T*_on_ = 410.7 ± 1.4 K) and Phe (*T*_on_ = 529.4 K), and (ii) as the Phe content per formula unit increases the fusion temperature becomes closer to that of pure L-phenylalanine (i.e., MA:Phe_2_ has a higher fusion temperature than MA:Phe). The first observation is very common for bi-component crystals [44]. One related example is glycine maleate (MA:Gly), with *T*_on_ = 417.6 ± 1.3 K [27] in the range of the fusion temperatures of maleic acid (MA form I polymorph; *T*_fus_ = 411.3 ± 0.4 K) [27] and *α*-glycine (*T*_fus_ = 525.0 ± 0.6 K) [27]. The closer proximity of the thermal properties of MA:Phe_2_ from those of pure L-phenylalanine parallels the trend noted for density in Table 1.

The temperature and enthalpy of fusion of MA(cr I) in Table 2 (*T*_on_ = 410.7 ± 1.4 K, $\Delta_{\mathrm{fus}}H_{m}^{o}$ = 38.6 ± 0.6 kJ mol^−1^) were also in close agreement with the results of previous determinations for the same polymorph, namely, *T*_fus_ = 411.9 K [45], *T*_fus_ = 411.3 ± 0.4 K [27], and $\Delta_{\mathrm{fus}}H_{m}^{o}$ = 37.5 ± 0.1 kJ mol^−1^ (the latter value, which refers to the mean of three determinations with the sample melting at 411.3 ± 0.4 K, is only being reported in the present work).

The DSC curve obtained for Phe(cr I) (Figure 5a) evidences that fusion starts at *T*_on_ ~ 518 K and is accompanied by thermal decomposition. This is reflected by a complex peak with maxima at 527 and 537 K. As mentioned above, thermal decomposition was also signaled by the presence of a brownish material inside the crucible at the end of the experiment. The same complex melting/decomposition behavior was observed by Lu et al. [46], who reported peak maxima at 533 K, 547 K, and 563 K. The somewhat higher *T*_max_ may be due, at least in part, to the higher heating rate used by these authors (10 K∙min^−1^). The present observations are also consistent with previous TG-FTIR studies indicating that Phe starts to undergo pyrolysis at 515 K with the formation of CO_2_, NH_3_, H_2_O, and benzeneethanamine [47]. It may finally be pointed out that the DSC curve obtained in this work exhibited a slow departure from the baseline starting at ~473 K, which is due to sample sublimation as subsequently confirmed by experiments performed on a Stuart SMP3 melting point apparatus.

### 2.4. Solution Calorimetry

In the case of MA:Phe and MA:Phe_2_, reactions (2) and (3) can be written in a compact form as:

MA:Phe*_b_*(cr) → MA(cr I) + *b*Phe(cr I) (4)

where cr I refers to the form I maleic acid or L-phenylalanine precursors used in this work, with *b* = 1 for reaction (2) and *b* = 2 for reaction (3). The standard molar enthalpy of reaction (4), $\Delta_{r}H_{m}^{o}(4)$, at 298.15 K, for MA:Phe and MA:Phe_2_ was obtained from [27,31]:

(5) $$\Delta_{r}H_{m}^{o}(4)=\Delta_{\mathrm{sol}}H_{m}^{o}(6)-\Delta_{\mathrm{sol}}H_{m}^{o}(7)-b\Delta_{\mathrm{sol}}H_{m}^{o}(8)$$

where $\Delta_{\mathrm{sol}}H_{m}^{o}(6)$, $\Delta_{\mathrm{sol}}H_{m}^{o}(7)$, and $\Delta_{\mathrm{sol}}H_{m}^{o}(8)$ represent the standard molar enthalpies of the following processes, at 298.15 K, determined by solution calorimetry:

MA:Pheb(cr) + *n*H_2_O(l) → [MA + bPhe + *n*H_2_O](aq) (6)

MA(cr I) + *n*H_2_O(l) → [MA + *n*H_2_O](aq) (7)

(8) $$\mathrm{Phe}(\mathrm{cr}\;I)+\frac{1}{b}[\mathrm{MA}+nH_{2}O](\mathrm{sln})\to \;[\mathrm{Phe}+\frac{1}{b}\mathrm{MA}+\frac{n}{b}H_{2}O](\mathrm{aq})$$

In the previous equations, *n* is the amount of substance of water used in the experiments per 1 mol of dissolved solid compound. The obtained results are summarized in Table 3 and further details are given in the Supporting Information.

### 2.5. Lattice Enthalpies

**Scheme 1:**
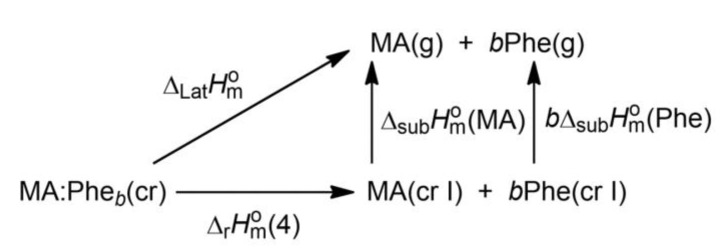
Thermodynamic cycle used to obtain the lattice enthalpy of the MA:Phe*_b_* bi-component crystals.

The lattice enthalpy is a useful concept to discuss the overall strength of all interactions responsible for the resilience of bi-component crystals towards dissociation. It can be defined as the standard molar enthalpy change, $\Delta_{\mathrm{Lat}}H_{m}^{o}$, associated with the disruption of the crystal lattice to originate the molecular components in the ideal gas state at a specific temperature (normally 0 K or 298.15 K) [26]. Scheme 1 shows that in the case of MA:Phe and MA:Phe_2_, $\Delta_{\mathrm{Lat}}H_{m}^{o}$, at 298.15 K, can be calculated from:

(9) $$\Delta_{\mathrm{Lat}}H_{m}^{o}({\mathrm{MA}:\mathrm{Phe}}_{b})=\Delta_{r}H_{m}^{o}(4)+\Delta_{\mathrm{sub}}H_{m}^{o}\left(\mathrm{MA}\right)+b\Delta_{\mathrm{sub}}H_{m}^{o}\left(\mathrm{Phe}\right)$$

where $\Delta_{r}H_{m}^{o}(4)$ is the enthalpy of reaction (4), $\Delta_{\mathrm{sub}}H_{m}^{o}$ denotes enthalpy of sublimation, and *b* is the stoichiometric coefficient of the Phe unit (*b* = 1 for MA:Phe and *b =* 2 for MA:Phe_2_). Based on $\Delta_{r}H_{m}^{o}(4)$ = 7.59 ± 0.20 kJ∙mol^−1^ for MA:Phe, $\Delta_{r}H_{m}^{o}(4)$ = 16.08 ± 0.27 kJ∙mol^−1^ for MA:Phe_2_ (Table 2), $\Delta_{\mathrm{sub}}H_{m}^{o}\left(\mathrm{MA}\right)$ = 110.0 ± 2.5 kJ∙mol^−1^ [48], and $\Delta_{\mathrm{sub}}H_{m}^{o}\left(\mathrm{Phe}\right)$ = 160.6 ± 3.5 kJ∙mol^−1^ [49], $\Delta_{\mathrm{Lat}}H_{m}^{o}(\mathrm{MA}:\mathrm{Phe})$ = 278.2 ± 4.3 kJ∙mol^−1^ and $\Delta_{\mathrm{Lat}}H_{m}^{o}({\mathrm{MA}:\mathrm{Phe}}_{2})$ = 447.3 ± 7.4 kJ∙mol^−1^ are obtained. The calculation of these lattice enthalpy values relies on the approximation that the $\Delta_{\mathrm{sub}}H_{m}^{o}$ data available in the literature for maleic acid and L-phenylalanine, which have no reference to specific crystal forms, can be assigned to the polymorphs used in this work.

The value $\Delta_{\mathrm{Lat}}H_{m}^{o}(\mathrm{MA}:\mathrm{Phe})$ = 278.2 ± 4.3 kJ∙mol^−1^ is significantly larger than that previously found for the analogous glycine maleate, $\Delta_{\mathrm{Lat}}H_{m}^{o}(\mathrm{MA}:\mathrm{Gly})$ = 257.5 ± 2.7 kJ∙mol^−1^ [27], despite the fact that the packing coefficient, density, and fusion temperature were smaller for the former (*k* = 0.695, *ρ* = 1.407 g cm^−3^, and *T*_on_ = 415.8 ± 0.6 K for MA:Phe) than for the latter (*k* = 0.743, *ρ* = 1.582 g cm^−3^, and *T*_on_ = 417.6 ± 1.3 K for MA:Gly). This shows that a larger lattice enthalpy does not necessarily imply a larger packing efficiency or fusion and thermal decomposition temperatures when different materials are compared.

**Figure 6:**
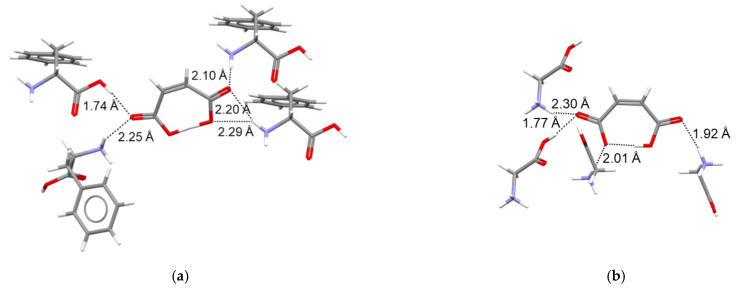
Hydrogen bond patterns around the maleate anion in (**a**) MA:Phe and (**b**) MA:Gly.

In addition, no clear link between the $\Delta_{\mathrm{Lat}}H_{m}^{o}(\mathrm{MA}:\mathrm{Phe})$ > $\Delta_{\mathrm{Lat}}H_{m}^{o}(\mathrm{MA}:\mathrm{Gly})$ relationship and the number and type of hydrogen bonds around the maleate anion center could be inferred. As shown in Figure 6, in both cases the MA^−^ anion is linked to four protonated amino acid molecules through one O−H···O and three N−H···O hydrogen bonds which, on average, are slightly shorter for MA:Gly (2.02 Å) than for MA:Phe (2.12 Å). Thus, the 20.7 kJ∙mol^−1^ larger lattice enthalpy of MA:Phe relative to that of MA:Gly should, most likely, reflect the increase in importance of van der Walls interactions and the presence of electrostatic interactions between aromatic rings, associated with the replacement of a hydrogen atom in glycine by a phenyl ring in L-phenylalanine. It is interesting to note that a similar difference (24.2 kJ∙mol^−1^) exists between the sublimation enthalpies of Phe (160.6 ± 3.5 kJ∙mol^−1^ [49]) and Gly (136.4 ± 0.4 kJ∙mol^−1^ [48]). This explains why the enthalpy of reaction (4) for MA:Phe in Table 2, $\Delta_{r}H_{m}^{o}(4)$ = 7.59 ± 0.20 kJ∙mol^−1^, differs only by 2.01 kJ∙mol^−1^ from the corresponding value reported for MA:Gly, $\Delta_{r}H_{m}^{o}(4)$ = 9.6 ± 1.0 kJ∙mol^−1^ [27].

### 2.6. Dissociation Enthalpies

The dissociation enthalpies of MA:Phe(cr) and MA:Phe_2_(cr) obtained from the $\Delta_{r}H_{m}^{o}(4)$ values in Table 3 are summarized in Table 4. Also included in Table 4 are the analogous data for the decomposition of glycine maleate to give MA(cr I) and γ-glycine, Gly(cr γ), which is the most stable glycine polymorph at 298.15 K [27]. An analysis of Table 4 shows that on enthalpic grounds:

(i) The MA:Phe_2_, MA:Phe, and MA:Gly bi-component crystals are stable towards dissociation into pure components, since positive $DH^{o}$ values were obtained in all cases;
(ii) MA:Phe_2_ should be more stable than MA:Phe, since $DH^{o}{{(\mathrm{MA}:\mathrm{Phe}}_{2})}_{\mathrm{cr}}$ = 2.1×$DH^{o}{\left(\mathrm{MA}:\mathrm{Phe}\right)}_{\mathrm{cr}}$. Given that $T_{\mathrm{fus}}({\mathrm{MA}:\mathrm{Phe}}_{2})$ = 444.6 ± 0.6 K was significantly larger than $T_{\mathrm{fus}}(\mathrm{MA}:\mathrm{Phe})$ = 415.8 ± 0.6 K (Table 2), this is in line with previous observations in bi-component co-crystals, suggesting that the stoichiometric combination with the higher melting point is the most stable (larger dissociation Gibbs energy) [24];
(iii) The first dissociation enthalpy of MA:Phe_2_, $DH_{1}^{o}{\left(A:B\right)}_{\mathrm{cr}}$= 8.49 ± 0.34 kJ mol^−1^, is only slightly larger than the second, $DH_{2}^{o}{\left(A:B\right)}_{\mathrm{cr}}$ = 7.59 ± 0.20 kJ mol^−1^. This indicates that the enthalpic cost of removing the zwitterionic Phe^±^ unit from MA:Phe_2_ to produce MA:Phe is not much different from that associated with the disruption of the MA:Phe lattice to yield neutral MA(cr I) and Phe(cr I), which implies the back neutralization of MA^−^ by proton transfer from HPhe^+^ and the concomitant rearrangement of the latter to a zwitterionic tautomer;
(iv) The dissociation enthalpy of MA:Phe (7.59 ± 0.20 kJ mol^−1^) is smaller than that of MA:Gly (9.6 ± 1.0 kJ mol^−1^), despite the fact that the opposite is observed in terms of lattice enthalpy (see Section 2.5). This stresses the fact that the $DH^{o}{\left(A:B\right)}_{\mathrm{cr}}$ values are not simply determined by the energetics of the bi-component crystal but also by that of the crystalline dissociation products considered.

## 3. Materials and Methods 

### 3.1. General Analytical Methods

Elemental C, H, and N analyses (EA) were performed on a Fisons EA1108 apparatus, with an uncertainty of ±0.3% for carbon and nitrogen and ±0.1% for hydrogen.

HPLC-ESI/MS analyses were carried out on an Ultimate RS system with a diode array detector coupled to an LCQ Fleet mass spectrometer (Thermo ScientificTM, CA, USA) equipped with an ESI ionization source. Chromatographic separation was achieved with a Phenomenex 100 Å C18 Luna^®^ column (150 × 4.6 mm i.d., 5 µm particle size), thermostated at 308.15 K. The mobile phase consisted of 0.1% formic acid in ultra-pure water (A) and acetonitrile (B). The elution gradient was as follows: 0 min, 5% B; 20 min, 70% B; 22 min, 100% B; 22–30 min 100% B; 30–45 min, 5% B. The injected sample volume was 10 μL, and the mobile phase flow was 300 μL min^−1^. Mass spectra obtained in positive ESI mode were acquired using the following optimized parameters: spray voltage = +4.5 kV, transfer capillary voltage = −18 V, ion transfer lens offset = +63 V, nebulizing gas (N_2_) pressure = 80 arbitrary units, drying gas (N_2_) pressure = 20 arbitrary units, transfer capillary temperature = 543 K. Data acquisition and processing were performed using Xcalibur 2.2 software.

Powder X-ray diffraction measurements were carried out at 296 ± 2 K on a D8 Advance Bruker diffractometer (Bruker, Karlsruhe, Germany) e quipped with a LinxEye detector. The radiation was produced with a Cu-Kα_1_ (λ = 1.5406 Å) tube, operated at 40 kV and 40 mA. The data collection was performed in the 2*θ* range 5–35° with a step size of 0.02°. Glass sample holders were used. The indexation of the powder patterns was performed with CellRef [50].

### 3.2. Materials

Maleic Acid (Fluka Analytical, ≥99.0 wt%) was used as received. Elemental analysis for C_4_H_4_O_4_: expected C 41.39%, H 3.47%; found C 41.31 ± 0.13%, H 3.37 ± 0.11% (average of two determinations; uncertainty corresponds to twice the mean deviation). The purity given by HPLC-ESI/MS analysis was >99.9%. The powder X-ray diffraction pattern was indexed as monoclinic, space group *P2_1_/c*, *a* = 7.481 ± 0.033 Å, *b* = 10.010 ± 0.015 Å, *c* = 7.620 ± 0.032 Å, *β* = 123.66 ± 0.28^o^ (see Supporting Information). This indicated that the sample corresponded to form I maleic acid, previously characterized by single-crystal X-ray diffraction (CSD Refcode: MALIAC11, monoclinic, space group *P2_1_*/*c*, *a* = 7.473 ± 0.001 Å, *b* = 10.098 ± 0.002 Å, *c* = 7.627 ± 0.002 Å, *β* = 123.59 ± 0.02^o^, [28,37]).

L-phenylalanine (Acros Organics, 98.5–101.0 wt%) was used as received. Elemental analysis for C_9_H_11_O_2_N: expected C 65.44%, H 6.71%, N 8.48%; found C 65.60 ± 0.52%, H 6.64 ± 0.18%, N 8.49 ± 0.08% (average of two determinations). The purity given by HPLC-ESI/MS analysis was >99.9%. The powder X-ray diffraction pattern was indexed as monoclinic space group *P2_1_*, *a* = 8.800 ± 0.012 Å, *b* = 6.042 ± 0.006 Å, *c* = 31.488 ± 0.035 Å, *β* = 96.58 ± 0.34^o^ (see Supporting Information). These results correspond to form I L-phenylalanine (CSD Refcode: QQQAUJ06, monoclinic, space group *P2_1_*, *a* = 8.7955 ± 0.0004 Å, *b* = 6.0363 ± 0.0002 Å, *c* = 31.5356 ± 0.0013 Å, *β* = 96.6441 ± 0.0014^o^ from single-crystal X-ray diffraction measurements [28,38]).

The MA:Phe and MA:Phe_2_ samples were prepared by mechanochemistry. Milling was performed with a Retsch MM400 apparatus and a 25 cm^3^ cylindrical stainless-steel jar containing two 5 mm diameter stainless-steel spheres. The frequency was set to 29 Hz and the grinding time was 15 min. The precursor materials were weighted with a precision of ±0.01 mg in a Mettler Toledo XS205 balance. In the case of MA:Phe, 0.08266 g (0.71 mmol) of maleic acid and 0.11770 g (0.71 mmol) of L-phenylalanine were used. Elemental analysis for C_13_H_15_NO_6_: expected C 55.51%, H 5.38%, N 4.98%; found C 55.51 ± 0.09%, H 5.34 ± 0.08%, N 4.96 ± 0.02% (average of two determinations). The powder pattern of the obtained product was indexed as monoclinic, space group *P2_1_*, *a* = 11.067 ± 0.010 Å, *b* = 5.342 ± 0.003 Å, *c* = 11.481 ± 0.010 Å, *β* = 101.10 ± 0.13° (see Supplementary Materials). These results are in agreement with the reported single-crystal X-ray diffraction data for the same material (CSD Refcode: EDAXIQ, monoclinic, space group *P2_1_*, *a* = 11.0560 ± 0.0009 Å, *b* = 5.3326 ± 0.0004 Å, *c* = 11.4712 ± 0.0007Å, *β* = 101.070 ± 0.010° [28,40]). MA:Phe_2_ was produced by griding 0.05174 g (0.45 mmol) of maleic acid with 0.14827 g (0.90 mmol) of L-phenylalanine. Elemental analysis for C_22_H_26_N_2_O_8_: expected C 59.19%, H 5.87%, N 6.27%; found C 58.75 ± 0.12%, H 5.95 ± 0.09%, N 6.23 ± 0.02% (average of two determinations). The powder pattern was indexed as monoclinic, space group *P2_1_*, *a* = 13.974 ± 0.020 Å, *b* = 5.425 ± 0.003 Å, *c* = 15.415 ± 0.022 Å, *β* = 108.78 ± 0.16° (see Supporting Information). These results are in excellent agreement with the corresponding data obtained in this work from single-crystal X-ray diffraction experiments (monoclinic, space group *P2_1_*, *a* = 13.967 ± 0.003 Å, *b* = 5.4188 ± 0.0009 Å, *c* = 15.398 ± 0.003 Å, *β* = 108.783 ± 0.005°, see details in Table 5).

### 3.3. Single-Crystal X-ray Diffraction

The selected single crystals were coated with Paratone-N oil and mounted in a Kapton loop. The single-crystal X-ray diffraction data were collected at 168 ± 2 K and 293 ± 2 K, using a BRUKER D8 QUEST (Bruker, Karlsruhe, Germany) diffractometer, with a PHOTON II detector or a Bruker AXS-KAPPA APEX II diffractometer. The radiation was produced by applying a potential difference and current of 50 kV and 30 mA, respectively, to a graphite-monochromated Mo-Kα source (λ = 0.71073 Å). The X-ray data collection was monitored by the Bruker APEX3 software [51]. An empirical absorption correction was enforced using Bruker SADABS [52], and data reduction was performed with Bruker SAINT [53]. The structures were solved by intrinsic phasing with Bruker SHELXT-2014 [54] and refined by full-matrix least-squares on *F*^2^ using SHELXL-2014/7 [54], both programs are included in WINGX-Version 2018.3 [55]. Atoms were refined with anisotropic thermal parameters. Most of the hydrogen atoms were inserted in calculated positions and allowed to refine in the parent carbon atom. The exception were those involved in hydrogen bonding to nitrogen or oxygen atoms, which were found in the difference electron density map. PLATON [43], also included in the WINGX program, was used for the hydrogen bond (H-bond) interactions and packing index. Structural representations were made with Mercury 2020.3.0 [41]. A summary of the crystal data, structure solution, and refinement parameters for the structures is given in Table 5.

### 3.4. Thermal Analysis

Thermogravimetry analyses were performed on a Perkin Elmer (Norwalk, CT, USA) TGA-7 apparatus. The samples with 2–15 mg mass were contained in a platinum crucible and studied at a heating rate of 5 K min^−1^ in the temperature range 298–618 K. The sample and balance chambers were purged with nitrogen gas (Praxair 5.0, 99.999%), with flows of 23 cm^3^∙min^−1^ and 38 cm^3^∙min^−1^, respectively. The mass scale of the apparatus was calibrated using a 100 mg standard mass. The calibration of the temperature scale was based on the Curie temperatures (*T*_C_) of nickel (Perkin–Elmer, 99.99%, *T_C_* = 628.45 K) and alumel (Perkin–Elmer, *T*_C_ = 427.35 K).

The DSC experiments were made on a Perkin Elmer (Norwalk, CT, USA) DSC-7. The samples with 1–7 mg mass were sealed inside aluminum crucibles and weighted with a precision of ±0.1 µg on a Mettler XP2U ultra-micro balance. The studies were carried out under a 30 cm^3^ min^−1^ flow of nitrogen (Praxair 5.0, 99.999%), in the temperature range 298–570 K, and at a heating rate of 5 K min^−1^. The temperature scale of the instrument was calibrated at the same heating rate by measuring the onset temperatures (*T*_on_) for the fusion of indium (Perkin–Elmer, 99.999%, *T*_on_ = 429.75 K) and zinc (Perkin–Elmer, 99.999%, *T*_on_ = 692.65 K). The calibration of the energy scale was based on the enthalpy of fusion of indium (∆_fus_ *h* = 28.45 J∙g^−1^).

The DSC and TG apparatus were both controlled by the Perkin Elmer (Shelton, CT, USA) Pyris V. 7.0.0.0110 software, which was also used for data acquisition and analysis.

### 3.5. Solution Calorimetry

Enthalpies of solution were determined at 298.15 K on a LKB 2277 Thermal Activity Monitor, using an in-house designed calorimetric cell made of stainless steel. The cell consists of a 20 cm^3^ cylindrical vessel closed by a lid that supports the sample drop system, the stirrer, and the calibration resistance. In a typical experiment, a pellet of sample with 10–15 mg mass, was weighted with a precision of ±0.1 µg on a Mettler XP2U ultra-micro balance and placed in the drop system. Approximately 13 cm^3^ of solvent were added to the cell body and weighted with a precision of ±0.01 mg using a Mettler XS 205 balance. The calorimetric cell was assembled and transferred to the calorimeter. Stirring (80 rpm) was started and, after an appropriate base line was acquired, the sample was dropped into the solvent. The molar enthalpy of the dissolution process, $\Delta_{\mathrm{sln}}H_{m}^{o}$, was obtained from:

(10) $$\Delta_{\mathrm{sln}}H_{m}^{o}=\frac{M}{m}\varepsilon (A-A_{b})$$

where *m* and *M* are the mass and molar mass of sample, respectively, *A* is the area of the observed calorimetric curve, $A_{b}$ is the contribution for the overall area from the activation of the drop mechanism, and *ε* is the calibration constant. The value of *A*_b_ = −(0.978 ± 0.072) mV·s was determined from a set of five experiments, where the drop mechanism was activated without a sample. The constant, *ε*, was obtained from a series of calibration experiments where a potential difference, *V*, was applied to 20 Ω resistance placed inside the calorimetric cell, causing a current of intensity, *I,* to flow through the resistance during a pre-programed time, *t*. This resulted in the dissipation of an amount of heat, *Q* = *VIt*, by Joule effect, which was reflected by a curve of area, *A*_cal_. The value of *ε* was calculated from:

(11) $$\varepsilon =\frac{\sum_{i}V_{i}I_{i}\Delta t_{i}}{A_{\mathrm{cal}}}$$

where *V_i_* and *I_i_* are the voltage and current intensity at a given time *t_i_*, respectively, and ∆*t_i_*~1 s is the time difference between two consecutive readings.

The control of the experiment and data acquisition were performed with the CBCAL 3.0 program [56]. EasyGraph II was used for data analysis [57].

## 4. Conclusions

Two bi-component crystals with different stoichiometries, MA:Phe and MA:Phe_2_, were synthesized from maleic acid (form I) and L-phenylalanine (form I), using mechanochemistry and characterized from structural and energetics points of view. Single-crystal X-ray diffraction (SCXRD) analysis showed that they are best described as organic salts: MA:Phe_2_ includes a maleate anion (MA^−^) and two L-phenylalanine units, one protonated (HPhe^+^) and another zwitterionic (Phe^±^); MA:Phe contains only MA^−^ and HPhe^+^ ions. In the first case, the main H-bond motif between the molecular units is of the N−H···O type and in the second N−H···O and O−H···O hydrogen bonds are present.

Calorimetric determinations showed that on enthalpic grounds: (i) MA:Phe and MA:Phe_2_ are both stable relative to dissociation into their precursors; (ii) the enthalpic cost of removing Phe^±^ from MA:Phe_2_ to yield MA:Phe (the first dissociation energy of MA:Phe_2_) is only 0.9 ± 0.4 kJ mol^−1^ larger than the cost of removing HPhe^+^ from MA:Phe (the second dissociation energy of MA:Phe_2_ which coincides with the dissociation enthalpy of MA:Phe); (iii) the latticed enthalpy difference between the two bi-component crystals, $\Delta_{\mathrm{Lat}}H_{m}^{o}({\mathrm{MA}:\mathrm{Phe}}_{2})$ − $\Delta_{\mathrm{Lat}}H_{m}^{o}(\mathrm{MA}:\mathrm{Phe})$= 169.1 ± 8.6 kJ mol^−1^, is comparable with the sublimation enthalpy of L-phenylalanine, $\Delta_{\mathrm{sub}}H_{m}^{o}(\mathrm{Phe})$ = 160.6 ± 3.5 kJ mol^−1^, indicating that, at least in this case, $\Delta_{\mathrm{Lat}}H_{m}^{o}$ is approximately additive.

Finally, the comparison of MA:Phe with the previously reported glycine maleate (MA:Gly), in terms of structure and energetics, suggested that structural/volumetric descriptors, such as packing coefficient, density, and hydrogen bond patterns, may not appropriately reflect dissociation enthalpy or lattice enthalpy trends in families of bi-component crystals differing in one of the co-formers, even if the stoichiometry is the same.

## Figures and Tables

**Table 1:** Unit cell parameters, density (*ρ*), molecular volume (*V*_o_), and packing coefficient (*k* = *ZV*_o_/*V*) for the crystal forms studied in this work *^a^*.

	MA(cr I) *^**b**^*	Phe(cr I) *^**c**^*	MA:Phe *^**d**^*	MA:Phe_2_ *^**e**^*
Space Group	*P*2_1_*/c*	*P*2_1_	*P*2_1_	*P*2_1_
*a*/Å	7.473 (1)	8.795 (4)	11.0560 (9)	13.9549 (17)
*b*/Å	10.098 (2)	6.0363 (2)	5.3326 (4)	5.4179 (6)
*c*/Å	7.627 (2)	31.5233 (13)	11.4712 (7)	15.4056 (16)
*β*/deg	123.59 (2)	96.6441 (12)	101.070 (10)	108.795 (10)
*V*/Å^3^	479.44 (17)	1662.40 (12)	663.73 (8)	1102.65 (21)
*Z*	4	8	2	2
*ρ*/g⋅cm^−3^	1.6081 (6)	1.3201 (1)	1.4073 (2)	1.3447 (3)
*V*_o_/Å^3^	86.7	144.2	230.6	377.7
*k*	0.723	0.694	0.695	0.685

*^a^* The *k* values were calculated with PLATON [43] and the corresponding *V*_o_ values were obtained as *V*_o_ = *kV*/*Z*. *^b^* CSD Refcode MALIAC11 [28,37]; *^c^* CSD Refcode QQQAUJ06 [28,38]; *^d^* CSD Refcode EDAXIQ [28,40]. *^e^* This work.

**Table 2:** Results of the differential scanning calorimetry (DSC) experiments on MA(cr I), Phe(cr I), MA:Phe, and MA:Phe_2_ *^a^*.

	Compound			
	MA(cr I)	Phen(cr I)	MA:Phe	MA:Phe_2_
*T*_on_/K	410.7 ± 1.4 (4)	517	415.8 ± 0.6 (12)	444.6 ± 0.6 (5)
*T*_max_/K	412.9 ± 1.0 (4)	527 (1) 537 (1)	418.8 ± 0.8 (12)	447.3 ± 0.8 (5)
$\Delta_{\mathrm{fus}}H_{m}^{o}$/kJ mol^−1^	38.6 ± 0.6	Dec.	Dec.	Dec.

*^a^T*_on_ and *T*_max_ are the temperatures corresponding to the onset and maximum of the DSC peak, respectively, (two maxima were observed in the case of Phe, see Figure 5a); $\Delta_{\mathrm{fus}}H_{m}^{o}$ is the enthalpy of fusion obtained by DSC when thermal decomposition (Dec.) did not occur. The assigned uncertainties were twice the standard error of the mean of the number of determinations given in parenthesis.

**Table 3:** Enthalpies of solution and reaction at 298.15 K. Data in kJ∙mol^−1^.

	Compound	
	MA:Phe	MA:Phe_2_
*b*	1	2
$\Delta_{r}H_{m}^{o}(6)$	34.97 ± 0.13	50.48 ± 0.22
$\Delta_{r}H_{m}^{o}(7)$	20.34 ± 0.15	20.34 ± 0.15
$\Delta_{r}H_{m}^{o}(8)$	7.04 ± 0.05	7.03 ± 0.06
$\Delta_{r}H_{m}^{o}(4)$	7.59 ± 0.20	16.08 ± 0.27

**Table 4:** Dissociation enthalpies at 298.15 K for MA:Phe_2_, MA:Phe, and MA:Gly.

Reaction	$DH_{\mathrm{cr}}^{o}{/\mathrm{kJ}\;\mathrm{mol}}^{-1}$
MA:Phe_2_(cr) → MA:Phe(cr) + Phe(cr I)	8.49 ± 0.34
MA:Phe(cr) → MA(cr I) + Phe(cr I)	7.59 ± 0.20
MA:Phe_2_(cr) → MA(cr I) + 2Phe(cr I)	16.08 ± 0.27
MA:Gly(cr) → MA(cr I) + Gly(cr γ)	9.6 ± 1.0 *^a^*

*^a^* Reference [27].

**Table 5:** Crystal data and structure refinement parameters for MA:Phe_2_.

CCDC reference number	2102636	2102637
Empirical formula	C_22_H_26_N_2_O_8_	C_22_H_26_N_2_O_8_
Formula weight	446.46	446.46
*T*/K	168 ± 2	293 ± 2
Crystal size/mm	0.40 × 0.35 × 0.10	0.80 × 0.60 × 0.20
Crystal color	Colorless	Colorless
Crystal system	Monoclinic	Monoclinic
Space group	*P*2_1_	*P*2_1_
*a*/Å	13.7482 (17)	13.9549 (17)
*b*/Å	5.4010 (7)	5.4179 (6)
*c*/Å	15.3787 (19)	15.4056 (16)
*β*/deg	109.028 (4)	108.795 (3)
*V*/Å^3^	1079.5 (2)	1102.7 (2)
*Z*/*Z*’	2/1	2/1
*ρ*_calc_/g⋅cm^−3^	1.373 (1)	1.345 (1)
*µ*/mm^−1^	0.105	0.103
*F* (000)	472	472
*θ* limits/deg	2.419–26.466	2.390–26.477
Limiting indices	–17 ≤ *h* ≤17	–17 ≤ *h* ≤17
	–6 ≤ *k* ≤ 6	–6 ≤ *k* ≤ 6
	–19 ≤ *l* ≤ 19	–19 ≤ *l* ≤ 19
Number of reflections collected/unique	17895/4434; [*R*(int) = 0.0599]	48992/4451; [*R*(int) = 0.0519]
Completeness to *θ*	99.7%	96.9%
Data/restraints/parameters	4434/1/321	4451/1/321
GOF on *F*^2^	1.067	1.054
Final *R* indices [*l* > 2σ(*l*)]	*R*_1_ = 0.0497; *R*_2_ = 0.1116	*R*_1_ = 0.0368; *R*_2_ = 0.0844
*R* indices (all data)	*R*_1_ = 0.0631; *R*_2_ = 0.1159	*R*_1_ = 0.0439; *R*_2_ = 0.0878
Largest diff peak and hole/*e* Å^3^	0.302 and −0.211	0.149 and –0.122

## Supplementary Materials

The following are available online: Tables S1–S4. The indexation of the powder patterns of the samples used in this work; Tables S5–S9. The solution calorimetry results; Figure S1: X-ray powder diffraction data suggesting that MA:Phe is an intermediate of MA:Phe_2_ formation by mechanochemistry; Figure S2: Comparison of the packing motifs in the reported MA:Phe structures at 293 K and 25 K; Figure S3. Enthalpy of solution of maleic acid in water as a function of the H_2_O/MA molar ratio, CIF files with the single-crystal X-ray diffraction structures of MA:Phe_2_ deposited at the Cambridge Crystallographic Data Center (CCDC) with reference numbers 2102636 (168 K) and 2102637 (293 K).

## Sample Availability

Samples of the compounds … are available from the authors.
