# Supplementary material for: First and Second Dissociation Enthalpies in Bi-Component Crystals Consisting of Maleic Acid and L-Phenylalanine
Source: Molecules. 2021 Sep 21;26(18):5714. doi: 10.3390/molecules26185714 (PMC8469174; doi:10.3390/molecules26185714)

checkCIF/PLATON report

Structure factors have been supplied for datablock(s) shelx

THIS REPORT IS FOR GUIDANCE ONLY. IF USED AS PART OF A REVIEW PROCEDURE FOR PUBLICATION, IT SHOULD NOT REPLACE THE EXPERTISE OF AN EXPERIENCED CRYSTALLOGRAPHIC REFEREE.

No syntax errors found. CIF dictionary Interpreting this report

Datablock: shelx

Bond precision: C-C = 0.0062 A Wavelength=0.71073

Cell:	a=13.7482(17)	b=5.4010(7)	c=15.3787(19)
	alpha=90	beta=109.028(4)	gamma=90
Temperature:	168 K		

	Calculated	Reported
Volume	1079.5(2)	1079.5(2)
Space group	P 21	P 21
Hall group	P 2yb	P 2yb
Moiety formula	C9 H12 N O2, C9 H11 N O2, C4 H3 O4	?
Sum formula	C22 H26 N2 O8	C22 H26 N2 O8
Mr	446.45	446.45
Dx,g cm-3	1.373	1.373
Z	2	2
Mu (mm-1)	0.105	0.105
F000	472.0	472.0
F000'	472.27	
h,k,lmax	17,6,19	17,6,19
Nref	4464[2480]	4434
Tmin,Tmax	0.959,0.990	0.514,0.745
Tmin'	0.959	

Correction method= # Reported T Limits: Tmin=0.514 Tmax=0.745
AbsCorr = MULTI-SCAN

Data completeness= 1.79/0.99 Theta(max)= 26.466

R(reflections)= 0.0497(3760) wR2(reflections)= 0.1159(4434)

S = 1.067 Npar= 321

The following ALERTS were generated. Each ALERT has the format
test-name_ALERT_alert-type_alert-level.
Click on the hyperlinks for more details of the test.

Alert level B

```
PLAT355_ALERT_3_B Long   O-H (X0.82,N0.98A)  O6       - H6O      .       1.08 Ang.
```

Alert level C

```
ABSTY02_ALERT_1_C  An _exptl_absorpt_correction_type has been given without
            a literature citation. This should be contained in the
            _exptl_absorpt_process_details field.
            Absorption correction given as multi-scan
STRVA01_ALERT_4_C           Flack parameter is too small
           From the CIF: _refine_ls_abs_structure_Flack   -1.200
           From the CIF: _refine_ls_abs_structure_Flack_su    0.600
PLAT222_ALERT_3_C NonSolvent Resd 1  H   Uiso(max)/Uiso(min) Range        8.1 Ratio
PLAT245_ALERT_2_C U(iso) H1NB      Smaller than U(eq) N1        by      0.016 Ang**2
PLAT245_ALERT_2_C U(iso) H1NC      Smaller than U(eq) N1        by      0.014 Ang**2
PLAT250_ALERT_2_C Large U3/U1 Ratio for Average U(i,j) Tensor ....        2.4 Note
PLAT340_ALERT_3_C Low Bond Precision on  C-C Bonds ..............    0.00624 Ang.
PLAT772_ALERT_2_C Suspect O-H Bond in CIF: O7       --H6O       ..       1.36 Ang.
PLAT911_ALERT_3_C Missing FCF Refl Between Thmin & STh/L=    0.600           4 Report
```

Alert level G

```
PLAT032_ALERT_4_G Std. Uncertainty on Flack Parameter Value High .      0.600 Report
PLAT303_ALERT_2_G Full Occupancy Atom H3O       with # Connections       2.00 Check
PLAT720_ALERT_4_G Number of Unusual/Non-Standard Labels ..........          6 Note
PLAT791_ALERT_4_G Model has Chirality at C12         (Sohnke SpGr)          S Verify
PLAT791_ALERT_4_G Model has Chirality at C15         (Sohnke SpGr)          S Verify
PLAT883_ALERT_1_G No Info/Value for _atom_sites_solution_primary .     Please Do !
PLAT910_ALERT_3_G Missing # of FCF Reflection(s) Below Theta(Min).          3 Note
PLAT912_ALERT_4_G Missing # of FCF Reflections Above STh/L=  0.600          7 Note
PLAT913_ALERT_3_G Missing # of Very Strong Reflections in FCF ....          2 Note
PLAT916_ALERT_2_G Hooft y and Flack x Parameter Values Differ by .       0.10 Check
PLAT961_ALERT_5_G Dataset Contains no Negative Intensities .......     Please Check
PLAT978_ALERT_2_G Number C-C Bonds with Positive Residual Density.          1 Info
```

0 **ALERT level A** = Most likely a serious problem - resolve or explain
1 **ALERT level B** = A potentially serious problem, consider carefully
9 **ALERT level C** = Check. Ensure it is not caused by an omission or oversight
12 **ALERT level G** = General information/check it is not something unexpected

2 ALERT type 1 CIF construction/syntax error, inconsistent or missing data
7 ALERT type 2 Indicator that the structure model may be wrong or deficient
6 ALERT type 3 Indicator that the structure quality may be low
6 ALERT type 4 Improvement, methodology, query or suggestion
1 ALERT type 5 Informative message, check

It is advisable to attempt to resolve as many as possible of the alerts in all categories. Often the minor alerts point to easily fixed oversights, errors and omissions in your CIF or refinement strategy, so attention to these fine details can be worthwhile. In order to resolve some of the more serious problems it may be necessary to carry out additional measurements or structure refinements. However, the purpose of your study may justify the reported deviations and the more serious of these should normally be commented upon in the discussion or experimental section of a paper or in the "special_details" fields of the CIF. checkCIF was carefully designed to identify outliers and unusual parameters, but every test has its limitations and alerts that are not important in a particular case may appear. Conversely, the absence of alerts does not guarantee there are no aspects of the results needing attention. It is up to the individual to critically assess their own results and, if necessary, seek expert advice.

Publication of your CIF in IUCr journals

A basic structural check has been run on your CIF. These basic checks will be run on all CIFs submitted for publication in IUCr journals (*Acta Crystallographica*, *Journal of Applied Crystallography*, *Journal of Synchrotron Radiation*); however, if you intend to submit to *Acta Crystallographica Section C* or *E* or *IUCrData*, you should make sure that full publication checks are run on the final version of your CIF prior to submission.

Publication of your CIF in other journals

Please refer to the *Notes for Authors* of the relevant journal for any special instructions relating to CIF submission.

PLATON version of 13/07/2021; check.def file version of 13/07/2021

Datablock shelx - ellipsoid plot

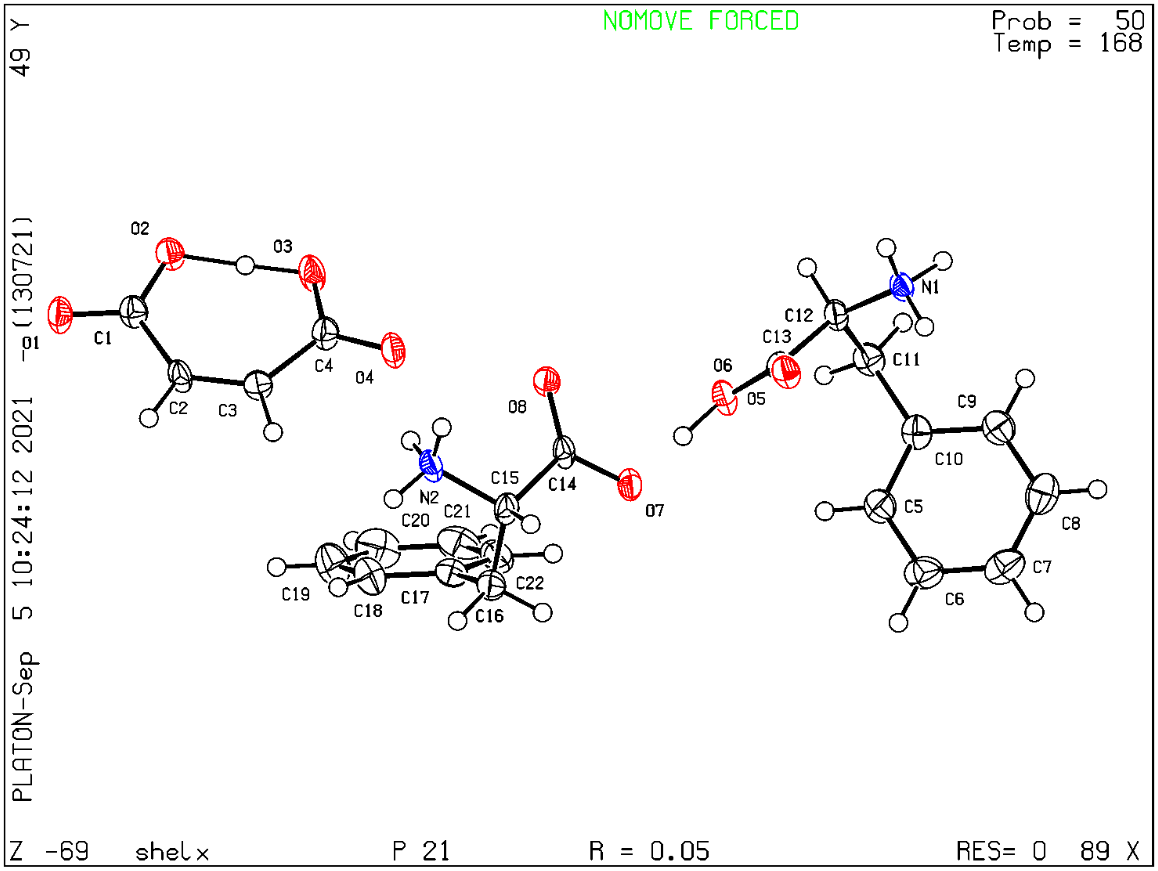

Supplement: Supplementary file 1 [file molecules-26-05714-s001.zip › MA-Phe2_168K_checkcif_R1.pdf]
