# Supplementary material for: First and Second Dissociation Enthalpies in Bi-Component Crystals Consisting of Maleic Acid and L-Phenylalanine
Source: Molecules. 2021 Sep 21;26(18):5714. doi: 10.3390/molecules26185714 (PMC8469174; doi:10.3390/molecules26185714)
Supplement: Supplementary file 1 [file molecules-26-05714-s001.zip › molecules-1362123_SI_R1.pdf]

molecules

Supporting Information

First and Second Dissociation Enthalpies of Bi-Component Crystals Consisting of Maleic Acid and L-Phenylalanine

Inês O. Feliciano [1,2], Daniela P. Silva [3], M. Fátima M. Piedade [2,3], Carlos E. S. Bernardes,[1,2] and Manuel E. Minas da Piedade [1,2,*]

1 Centro de Química Estrutural, Faculdade de Ciências, Universidade de Lisboa, 1749-016 Lisboa, Portugal; idfeliciano@fc.ul.pt; cebernardes@fc.ul.pt; mdpiedade@fc.ul.pt; memp@fc.ul.pt
2 Departamento de Química e Bioquímica, Faculdade de Ciências, Universidade de Lisboa, 1749-016 Lisboa, Portugal
3 Centro de Química Estrutural, Instituto Superior Técnico, Universidade de Lisboa, 1049-001 Lisboa, Portugal; daniela.filipa.2710@hotmail.com; mdpiedade@fc.ul.pt

* Correspondence: memp@fc.ul.pt; Tel.: +351-217500005 (MEMP)

Contents:

S1. Results of Powder X-ray Diffraction (PXRD) Analyses 2

S2. Solution Calorimetry Results.. 4

S3. References... 6

S1. Results of Powder X-ray Diffraction (PXRD) Analyses

The results from the indexation of the powder patterns of the samples used in this work are summarized in Tables S.1 to S.4, where $\Delta 2\theta$ denotes the difference between the 2θ values for peaks experimentally observed and those expected based on the indexed unit cell parameters.

Figure S1 shows a comparison of diffractograms obtained at different time points along the mechanochemical synthesis of MA:Phe_2, with PXRD patterns corresponding to pure, maleic acid (MA, form I), L-phenylalanine (Phe, form I), MA:Phe, and MA:Phe_2. The results indicate that after 5 min the reaction mixture (initially consisting of MA and Phe in 1:2 molar ratio) already contains MA:Phe and MA:Phe_2. The amounts of MA, Phe and MA:Phe progressively decrease with the griding time (see blue, green, and orange dash lines) and, at 20 min, complete conversion to MA:Phe_2 has occurred. These observations suggest that MA:Phe is a precursor of MA:Phe_2.

Table S1. Indexation of the powder X-ray diffraction pattern of maleic acid (form I) in the range $5^o \leq 2\theta \leq 35^o$ (space group $P2_1/c$; a = 7.481±0.033 Å, b = 10.010 ± 0.015 Å, c = 7.620±0.032 Å, β = 123.66±0.28°).

h	k	l	2θ/°	$\Delta 2\theta$/ °
1	1	0	16.765	0.040
0	2	0	17.579	0.017
−1	2	1	22.090	−0.007
0	2	1	22.509	0.004
−1	1	2	24.986	−0.024
−2	1	1	25.373	−0.084
−2	0	2	26.856	0.064
0	0	2	28.149	0.013
−1	2	2	29.384	−0.008
1	2	1	30.698	−0.003
−2	2	2	32.214	−0.003
0	2	2	33.340	−0.021
2	2	0	33.847	0.027

Table S2. Indexation of the powder X-ray diffraction pattern of L-phenylalanine (form I) in the range 5° ≤ 2θ ≤ 35° (space group $P2_1$, a = 8.800±0.012 Å, b = 6.042±0.006 Å, c = 31.488±0.035 Å, β = 96.58±0.34°).

h	***k***	***l***	**2*θ*/°**	**Δ2*θ*/ °**	***h***	***k***	***l***	**2*θ*/°**	**Δ2*θ*/ °**
0	0	2	5.645	−0.001	−1	1	6	23.891	−0.007
0	0	4	11.307	0.001	0	1	7	24.733	−0.027
0	1	2	15.702	−0.008	0	0	9	25.677	0.067
0	0	6	16.989	−0.005	2	1	2	26.317	0.025
1	1	0	17.829	−0.002	0	1	8	27.159	0.017
1	0	5	18.330	−0.015	0	0	10	28.491	−0.021
−1	1	3	19.240	−0.019	0	1	9	29.620	−0.008
1	1	3	20.285	0.014	2	1	5	30.058	−0.047
−1	1	4	20.521	−0.001	−3	0	3	30.866	−0.011
1	1	4	21.835	0.050	−1	1	10	32.787	−0.006
0	0	8	22.710	−0.014	−3	1	3	34.353	0.019

Table S3. Indexation of the powder X-ray diffraction pattern of MA:Phe in the range 5° ≤ 2θ ≤ 35° (space group $P2_1$, a = 11.067±0.010 Å, b = 5.342±0.003 Å, c = 11.481±0.010 Å, β = 101.10±0.13°).

h	*k*	*l*	2*θ*/°	Δ2*θ*/ °	*h*	*k*	*l*	2*θ*/°	Δ2*θ*/ °
1	0	0	8.186	0.045	1	1	2	25.361	−0.041
1	0	1	12.339	−0.022	2	1	1	25.674	−0.012
−1	0	2	16.248	−0.022	−2	1	2	26.426	0.004
−2	0	1	16.677	−0.026	0	1	3	29.048	−0.018
0	1	1	18.362	−0.019	−3	1	1	29.590	−0.009
1	0	2	19.070	−0.024	2	1	2	30.065	0.017
2	0	1	19.482	0.017	−2	1	3	31.116	0.009
−2	0	2	20.398	−0.015	2	0	3	31.485	0.021
1	1	1	20.729	−0.017	0	0	4	31.805	0.035
0	1	2	22.925	−0.019	3	1	1	32.114	0.007
−1	1	2	23.343	0.021	−4	0	0	32.991	0.001
−2	1	1	23.666	0.036	−1	2	1	35.167	0.019
−3	0	0	24.579	−0.012	2	1	3	35.768	−0.009

Table S4. Indexation of the powder X-ray diffraction pattern of MA:Phe2 in the range 5° ≤ 2*θ* ≤ 35° (space group *P*21, *a* = 13.974±0.020 Å, *b* = 5.425±0.003 Å, *c* = 15.415±0.022 Å, *β* = 108.78±0.16°).

h	*k*	*l*	2*θ*/°	Δ2*θ*/ °	*h*	*k*	*l*	2*θ*/°	Δ2*θ*/ °
1	0	0	6.662	−0.019	2	1	0	21.187	0.008
−1	0	2	11.789	−0.026	−2	1	2	22.176	−0.006
−2	0	1	12.770	−0.021	1	1	2	22.688	−0.006
−2	0	0	13.375	−0.009	−1	0	4	23.094	−0.013
−2	0	2	14.884	−0.018	−2	0	4	23.813	0.055
1	0	2	15.629	−0.014	0	1	3	24.570	−0.008
2	0	1	16.396	0.001	−2	1	3	25.125	0.085
0	1	1	17.429	−0.010	−4	0	1	25.630	0.003
−1	1	1	17.948	−0.023	3	1	0	26.041	0.004
0	0	3	18.227	−0.009	2	1	2	26.572	−0.011
−2	0	3	18.806	−0.039	4	0	0	26.967	0.012
−3	0	1	19.057	0.000	−3	1	3	27.861	−0.016
1	1	1	19.387	−0.002	−2	1	4	28.954	−0.016
−3	0	2	19.878	−0.012	0	0	5	30.638	0.010
3	0	0	20.167	0.033	−3	1	4	31.038	−0.005
0	1	2	20.404	0.007	4	1	0	31.673	−0.008
−2	1	1	20.814	0.010					

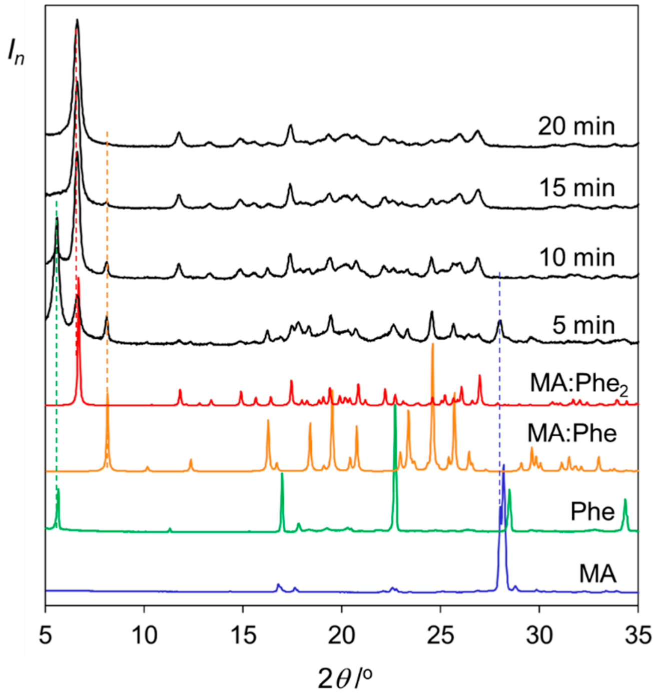

Figure S1. Comparison of diffractograms obtained for a 1:2 mixture of maleic acid (form I) and L-phenylalanine (form I), at different time points along the mechanochemical synthesis of MA:Phe2, with PXRD patterns corresponding to pure, maleic acid (form I), L-phenylalanine (form I), MA:Phe, and MA:Phe2. The dash lines refer to characteristic peaks of MA (blue), Phe (green), MA:Phe (orange) and MA:Phe2 (red).

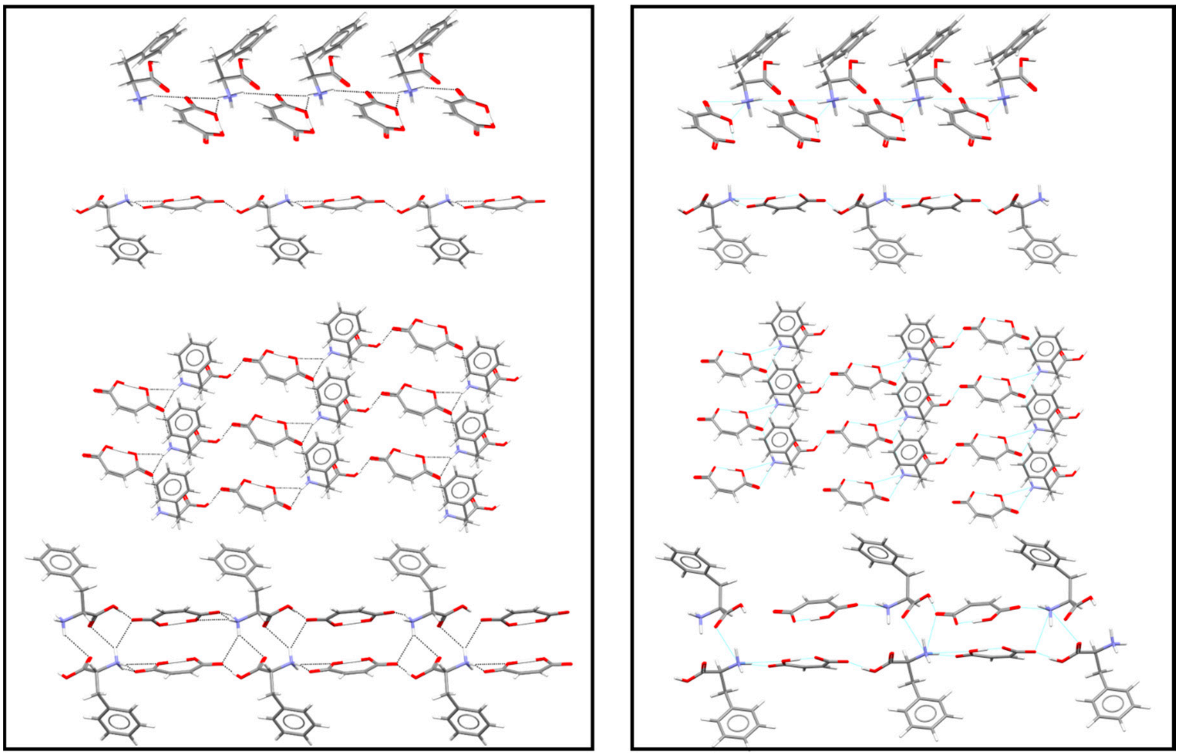

Figure S2. Comparison of the packing motifs in the reported MA:Phe structures at 293 K (left; CSD Refcode EDAXIQ) [1, 2] and 25 K (right; CSD Refcode EDAXIQ03 [2, 3]).

S2. Solution Calorimetry

The results of the solution calorimetry measurements corresponding to the processes:

$$\text{MA:Phe}_b\text{(cr)} + n\text{H}_2\text{O(l)} \rightarrow [\text{MA} + b\text{Phe} + n\text{H}_2\text{O}]\text{(sln)} \quad \text{(S1)}$$

$$\text{MA(cr)} + n\text{H}_2\text{O(l)} \rightarrow [\text{MA} + n\text{H}_2\text{O}]\text{(sln)} \quad \text{(S2)}$$

$$\text{Phe(cr)} + \frac{1}{b}[\text{MA} + n\text{H}_2\text{O}]\text{(sln)} \rightarrow [\text{Phe} + \frac{1}{b}\text{MA} + \frac{n}{b}\text{H}_2\text{O}]\text{(sln)} \quad \text{(S3)}$$

are listed in Tables S.5 to S.9, where m, $m_{\text{H}_2\text{O}}$, and m_{sln} are the masses of compound, water, and solution, respectively; A is the area of the calorimetric curve; $A_b = -0.978\pm0.072$ mV·s, is the area correction corresponding to the drop process; e if the calibration constant; n is the number of water molecules per mole of maleic acid in the mixture; and $\Delta_{\text{sol}}h^{\text{o}}$ is the standard specific enthalpy of solution.

The uncertainties assigned to the mean values of the standard specific enthalpies of solution, $<\Delta_{\text{sol}}h^{\text{o}}>$, are combined standard uncertainties, u_{c} [4], calculated as:

$$u_c = <\Delta_{\text{sol}}h^{\text{o}}> \sqrt{\left(\frac{u_{\Delta_{\text{sol}}h^{\text{o}}}}{<\Delta_{\text{sol}}h^{\text{o}}>}\right)^2 + \left(\frac{u_\varepsilon}{<\varepsilon>}\right)^2} \quad \text{(S4)}$$

where u_ε and $u_{\Delta_{\text{sol}}h^{\text{o}}}$ are the uncertainty contributions from the calibration and the main experiment, respectively, calculated as standard deviations of the mean [4]:

$$u = \sqrt{\frac{\sum_i^n \left(\Delta_{\text{sol}}h_i^{\text{o}} - <\Delta_{\text{sol}}h^{\text{o}}>\right)^2}{n(n-1)}} \quad \text{(S5)}$$

where $\Delta_{\text{sln}}h_i^{\text{o}}$ is the value corresponding to the ith measurement and n is the number of independent measurements.

All standard molar enthalpies of solution were calculated as:

$$\Delta_{\text{sln}}H_{\text{m}}^{\text{o}} = M <\Delta_{\text{sol}}h^{\text{o}}> \quad \text{(S6)}$$

where M is the molar mass of the reference compound calculated from the conventional standard atomic masses recommended by IUPAC in 2013 [5].The assigned uncertainties were taken as expanded uncertainties, U [4], given by:

$$U = 2Mu_{\text{c}} \quad \text{(S7)}$$

where u_{c} is uncertainty of the corresponding standard specific enthalpy of solution calculated from eq. S4.

Table S5. Solution calorimetry results for the dissolution of MA:Phe in water (eq. S1, *b* = 1).[a]

m/mg	m_{H_2O}/g	n	ε/μW mV^{-1}	$A-A_b$/mV s	$\Delta_{sol}h^{o}$ /J g^{-1}
27.2956	13.0660	7473.6	6.883	491.333	124.149
26.7129	13.0966	7654.5	6.913	483.012	124.709
26.7320	13.0169	7602.5	6.907	483.071	124.635
24.1415	13.0456	8436.9	6.879	432.806	123.649
26.7498	13.1351	7666.4	6.905	482.627	124.438

[a] $<\varepsilon>$ = 6.897±0.007 μW mV^{-1}; $<n>$ = 7767±171; $<\Delta_{sol}h^{o}>$ = 124.316±0.193 J g^{-1}; *M*(MA:Phe)= 281.264 g mol^{-1}; $\Delta_{sol}H^{o}_{m}$ = 34.97±0.13 kJ mol^{-1}

Table S6. Solution calorimetry results for the dissolution of MA:Phe_2 in water (eq. S1, *b* = 2)[a]

m/mg	m_{H_2O}/g	n	ε/μW mV^{-1}	$A-A_b$/mV s	$\Delta_{sol}h^{o}$ /J g^{-1}
42.8930	13.0501	7539.8	6.965	700.784	113.140
43.7776	12.9930	7355.1	6.934	713.197	112.818
42.7106	13.0329	7562.0	6.974	697.214	113.045
43.2841	13.0060	7446.4	6.945	707.024	113.116
43.2596	13.1014	7505.3	6.808	707.603	113.273

[a] $<\varepsilon>$ = 6.925±0.030 μW mV^{-1}; $<n>$ = 7482±37; $<\Delta_{sol}h^{o}>$ = 113.078±0.075 J g^{-1}; *M*(MA:Phe_2)= 446.456 g mol^{-1}; $\Delta_{sol}H^{o}_{m}$ = 50.48±0.22 kJ mol^{-1}

Table S7. Solution calorimetry results for the dissolution of maleic acid in water (eq. S2).[a]

m/mg	m_{H_2O}/g	n	ε/μW mV^{-1}	$A-A_b$/mV s	$\Delta_{sol}h^{o}$ /J g^{-1}
11.3096	13.04615	7432.4	7.164	275.633	173.867
10.8922	13.0362	7711.3	7.129	270.790	177.358
11.3384	13.05156	7416.6	7.131	278.323	175.118
11.7892	13.01253	7111.7	7.127	290.580	175.839
11.9902	13.07812	7027.7	7.121	292.685	174.143

[a] $<\varepsilon>$ = 7.134±0.008 μW mV^{-1}; $<n>$ = 7140±123; $<\Delta_{sol}h^{o}>$ = 175.265±0.630 J g^{-1}; *M*(MA)= 116.072 g mol^{-1}; $\Delta_{sol}H^{o}_{m}$ = 20.34±0.15 kJ mol$^{-1.}$

Table S8. Solution calorimetry results for the dissolution of L−phenylalanine in an aqueous solution of maleic acid (eq. S3, *b* = 1).[a]

m/mg	m_{sln}/g	n	ε/μW mV^{-1}	$A-A_b$/mV s	$\Delta_{sol}h^{o}$ /J g^{-1}
16.0727	13.1716	7425.9	6.657	103.639	42.867
14.0275	13.0256	7704.9	6.693	89.314	42.328
16.2460	13.0875	7410.1	6.612	104.087	42.593
15.8153	13.0093	7105.2	6.661	101.854	42.815
16.7553	13.0668	7021.2	6.617	107.056	42.477

[a] $<\varepsilon>$ = 6.648±0.015 μW mV^{-1}; $<n>$ = 7333±123; $<\Delta_{sol}h^{o}>$ = 42.616±0.101 J g^{-1}; *M*(Phe)= 165.192 g mol^{-1}; $\Delta_{sol}H^{o}_{m}$ = 7.04±0.05 kJ mol^{-1}

Table S9. Solution calorimetry results for the dissolution of L−phenylalanine in an aqueous solution of maleic acid (eq. S3; b = 2).[a]

m/mg	m_{sln}/g	n	ε/μW mV^{-1}	$A-A_b$/mV s	$\Delta_{\mathrm{sol}}h^{\mathrm{o}}$ /J g^{-1}
32.6086	12.9693	7427.9	6.635	206.980	42.261
31.8949	13.2786	7427.9	6.693	205.646	42.928
32.6640	13.2125	7427.9	6.644	208.383	42.475
32.2685	13.0757	7427.9	6.636	207.733	42.862
32.1997	13.3839	7427.9	6.683	204.248	42.233

[a] $\langle\varepsilon\rangle$ = 6.658±0.012 μW mV^{-1}; $\langle n\rangle$ = 7428; $\langle\Delta_{\mathrm{sol}}h^{\mathrm{o}}\rangle$ = 42.552±0.147 J g^{-1}; M(Phe)= 165.192 g mol^{-1}; $\Delta_{\mathrm{sol}}H^{\mathrm{o}}_{\mathrm{m}}$ = 7.03±0.02 kJ mol^{-1}

The standard molar enthalpy of solution of maleic acid in water (Table S7) had been previously reported for two different water/MA molar ratios (n) by Apelblat (n = 2162, $\Delta_{\mathrm{sol}}H^{\mathrm{o}}_{\mathrm{m}}$ = 21.333±0.029 kJ·mol^{-1}) [6] and Évora *et al.* (n = 9500, $\Delta_{\mathrm{sol}}H^{\mathrm{o}}_{\mathrm{m}}$ = 19.47±0.52 kJ·mol^{-1}) [7]. These results seem to be consistent with that obtained in this work (n = 7340, $\Delta_{\mathrm{sol}}H^{\mathrm{o}}_{\mathrm{m}}$ = 20.34±0.15 kJ·mol^{-1}, Table S7). Indeed, when the three independently determined $\Delta_{\mathrm{sol}}H^{\mathrm{o}}_{\mathrm{m}}$ values are plotted as a function of n (Figure S3) a least-squares fitting leads to the following linear relationship with a determination coefficient of R^2 = 0.962.

$$\Delta_{\mathrm{sol}}H^{\mathrm{o}}_{\mathrm{m}}(\mathrm{S2})\ /\mathrm{kJ{\cdot}mol^{-1}} = -(2.43\pm0.48)\times10^{-4}n + (21.92\pm0.34) \qquad \text{(S8)}$$

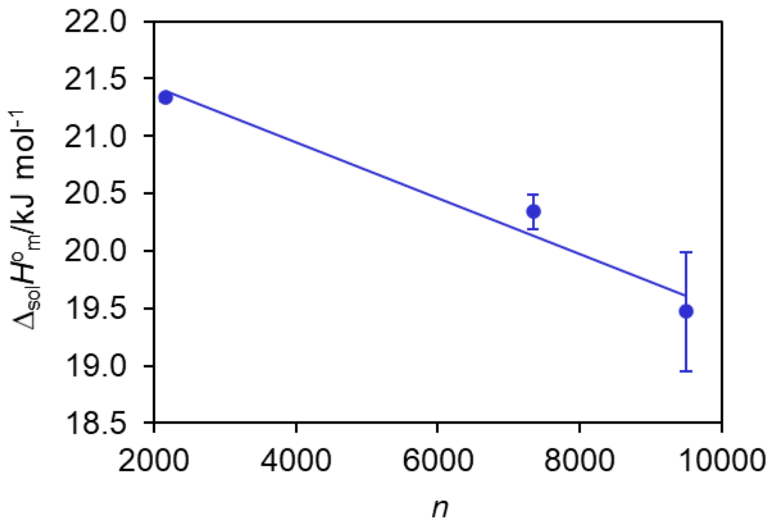

Figure S3. Enthalpy of solution of maleic acid in water as a function of the H_2O/MA molar ratio, n.

S3. References

1. Alagar, M.; Krishnakumar, R. V.; Natarajan, S., L-phenylalaninium maleate. *Acta Crystallogr.* **2001**, E57, o968-o970.
2. Groom, C. R.; Bruno, I. J.; Lightfoot, M. P.; Ward, S. C., The Cambridge Structural Database. *Acta Crystallogr.* **2016**, B72, 171-179.
3. Woińska, M.; Jayatilaka, D.; Spackman, M. A.; Edwards, A. J.; Dominiak, P. M.; Woźniak, K.; Nishibori, E.; Sugimoto, K.; Grabowsky, S., Hirshfeld atom refinement for modelling strong hydrogen bonds. *Acta Crystallogr.* **2014**, A70, 483-498.
4. Taylor, B. N.; Kuyatt, C. E., *Guidelines for evaluating and expressing the uncertainty of NIST measurements (NIST Technical Note 1297)*. NIST: Gaithersburg, 1994.
5. Meija, J.; Coplen, T. B.; Berglund, M.; Brand, W. A.; De Bièvre, P.; Gröning, M.; Holden, N. E.; Irrgeher, J.; Loss, R. D.; Walczyk, T.; Prohaska, T., Atomic weights of the elements 2013 (IUPAC Technical Report). *Pure Appl. Chem.* **2016**, 88, 265-291.
6. Apelblat, A., Enthalpy of solution of oxalic, succinic, adipic, maleic, malic, tartaric, and citric acids, oxalic-acid dihydrate, and citric-acid monohydrate in water at 298.15-K. *J. Chem. Thermodyn.* **1986**, 18, 351-357.
7. Évora, A. O. L.; Bernardes, C. E. S.; Piedade, M. F. M.; Conceição, A. C. L.; Minas da Piedade, M. E., Energetics of glycine cocrystal or salt formation with two regioisomers: Fumaric acid and maleic acid. *Cryst. Growth Des.* **2019**, 19, 5054-5064.
